# Extremely Early Appearance of Islet Autoantibodies in Genetically Susceptible Children

**DOI:** 10.1155/2023/9973135

**Published:** 2023-12-11

**Authors:** Anni Kyrönniemi, Toni Valtanen, Jaakko Koskenniemi, Paula Vähäsalo, Taina Härkönen, Jorma Ilonen, Jorma Toppari, Mikael Knip, Riitta Veijola

**Affiliations:** ^1^Department of Pediatrics, Research Unit of Clinical Medicine, Medical Research Center, University of Oulu, P.O. Box 5000, Oulu 90014, Finland; ^2^Department for Children and Adolescents, Medical Research Center, Oulu University Hospital, P.O. Box 10, Oulu 90029, Finland; ^3^Department of Pediatrics, Turku University Hospital, P.O. Box 52, Turku 20521, Finland; ^4^Institute of Biomedicine, Research Centre for Integrative Physiology and Pharmacology, and Centre for Population Health Research, University of Turku, 20014, Turku, Finland; ^5^Research Program for Clinical and Molecular Metabolism, Faculty of Medicine, University of Helsinki, P.O. Box 63, Helsinki 00014, Finland; ^6^Immunogenetics Laboratory, Institute of Biomedicine, University of Turku, 20014, Turku, Finland; ^7^Tampere Centre for Child Health Research, Tampere University Hospital, P.O. Box 2000, Tampere 33521, Finland; ^8^Pediatric Research Center, Children's Hospital, Helsinki University Hospital, P.O. Box 347, Helsinki 00029, Finland

## Abstract

**Objective:**

We studied the characteristics of children who developed islet autoantibodies by the age of 0.50 years and hypothesized that the appearance of extremely early islet autoimmunity differs between four birth cohorts within 1994–2019 according to the change in the incidence of Type 1 diabetes (T1D) in Finland.

**Methods:**

Data from Finnish children participating in the Type 1 Diabetes Prediction and Prevention (DIPP) study, or the Environmental Determinants of Diabetes in the Young (TEDDY) study were analyzed. These studies follow children with increased HLA-conferred risk for T1D with regular measurements of islet autoantibodies. Maternally transferred antibodies were excluded by comparing islet autoantibodies in cord serum, child's first follow-up serum and the maternal serum.

**Results:**

Among 20,979 Finnish children at increased risk to T1D, 53 (0.25%) developed at least one islet autoantibody at the age of ≤0.50 years. During a mean follow-up of 8.1 years, 15.1% progressed to T1D (median age at diagnosis 2.0 years), 43.4% developed confirmed islet autoimmunity but no T1D, and 41.5% had only transient islet autoantibodies. IAA was the most common first-appearing autoantibody. Among progressors, age at diagnosis was 1.0–2.4 years in children with IAA-initiated autoimmunity and 4.5–16.1 years in ZnT8A-initiated autoimmunity. When comparing children developing autoantibodies either at the age of ≤0.50 years or 0.51–0.75 years, confirmed positivity during follow-up was more common in the older group (81.7% vs. 58.5%; *p*=0.002). In four birth cohorts within 1994–2019 appearance of islet autoantibodies at the age of ≤0.50 years decreased towards the most recent birth cohorts (*p*=0.016).

**Conclusion:**

Islet autoimmunity by the age of 0.50 years was rare in genetically susceptible children and was typically initiated with IAA. Confirmed positivity was less common in children with autoantibodies at age ≤0.50 than at slightly older age. The secular decrease of islet autoimmunity before age 0.50 years was observed. This trial is registered with NCT03269084 and NCT00279318.

## 1. Introduction

Early appearance of islet autoantibodies is linked to rapid progression to Type 1 diabetes (T1D) [1, 2] and young age at diagnosis is associated with more severe symptoms and a high incidence of ketoacidosis [3]. The first 2 years of life represent the most common age of appearance of islet autoantibodies in children [4, 5]. The incidence of insulin autoantibodies (IAA) and autoantibodies against zinc transporter 8 (ZnT8A) as the first appearing islet autoantibodies reach their peak around 1 year of age, after which their appearance decreases, while the development of antibodies against glutamic acid decarboxylase (GADA) increases until the age of 3–5 years and remains rather stable for years [5–7]. IAA as the first appearing islet autoantibody at a young age is associated with a rapid progression to T1D [2].

The incidence of T1D in children has been rising worldwide, and the incidence is especially high in Finland [8, 9], where it has more than doubled from 1980 to 2006, remaining rather stable thereafter. The rise in incidence has been most prominent in the youngest children [10, 11].

Maternal islet antibodies can be transferred transplacentally to the fetus during pregnancy, and they can persist in the child's circulation up to the second year of life [12, 13]. Consequently, it may be challenging to determine the origin of islet autoantibodies detected in infancy and there is few information on children who develop islet autoantibodies under the age of 6 months. In the Norwegian Environmental Triggers of Type 1 Diabetes (MIDIA) study, two children out of 526 had developed islet autoantibodies before the age of 6 months [14] and among 8,503 children participating in the Environmental Determinants of Diabetes in the Young (TEDDY) study 10 had seroconverted by the age of 3 months and another 21 by the age of 6 months [5].

The role of extremely early appearance of islet autoimmunity in the T1D disease process has remained rather unestablished. The aim of this study was to investigate the frequency of islet autoimmunity at the age of ≤0.50 years and clinical characteristics of such individuals among genetically susceptible Finnish children participating in the Type 1 Diabetes Prediction and Prevention (DIPP) or TEDDY study. We also explored whether the proportion of children with extremely early autoimmunity has changed according to the incidence of T1D in Finland.

## 2. Subjects and Methods

### 2.1. Study Design

The Finnish DIPP study and international TEDDY study are prospective follow-up studies of children with HLA class II conferred risk to T1D. The DIPP study started in Finland in the mid 1990s. The TEDDY study recruited Finnish children with the highest HLA class II risk from September 2004 to February 2010. In three Finnish delivery hospitals (Oulu, Tampere, and Turku), the parents of every newborn baby were informed about the studies and asked to sign a written consent to screen the cord blood sample of the baby for HLA-associated genetic susceptibility to T1D. Children with increased HLA-conferred risk were invited to the DIPP follow-up, but during TEDDY recruitment period children with the highest HLA-conferred risk were invited to the TEDDY follow-up. The first follow-up visits in both studies were scheduled at ages 3 and 6 months and thereafter at 3–12 months intervals until the age of 15 years or diagnosis of T1D. For children who developed islet autoantibodies the subsequent visit frequency was every 3 months [6, 15].

The DIPP study was originally approved by the Ethics Committees of the regional university hospitals, and since 2018 by the Ethics Committee of the Hospital District of Northern Ostrobothnia. The TEDDY study was approved by Institutional Review or Ethics Boards in each participating center, in Finland by the Ethics Committee of the Hospital District of Southwest Finland, and an External Evaluation Committee formed by the National Institutes of Health has monitored the study [2].

The data of the participants in the TEDDY study were received from the National Institute of Diabetes and Digestive and Kidney Diseases (NIDDK) Central Repository in May 2022. The data were processed according to the Data Use Agreement signed by both parties.

### 2.2. Islet Autoantibodies in the DIPP Study

Islet autoantibody data until April 2022 were used in the current analysis. GADA, IAA, insulinoma-associated antigen-2 autoantibodies (IA-2A), and ZnT8A were analyzed with specific radio binding techniques as described earlier [16–19]. Islet cell autoantibodies (ICA) were determined with indirect immunofluorescence [20]. ICA, IAA, GADA, and IA-2A were analyzed in the Diabetes Research Laboratory, Department of Pediatrics, University of Oulu. ZnT8A were analyzed in the PEDIA Laboratory, University of Helsinki until July 2021, and thereafter the Diabetes Research Laboratory, Department of Pediatrics, University of Oulu. The performance of the assays was regularly assessed in the Diabetes Islet Autoantibody Standardization Program (DASP) and Islet Autoantibody Standardization Program (IASP). In 2002–2020 the sensitivity and specificity of the assays in these workshops were 36%–58% and 96%–99% for IAA, 60%–88% and 95%–100% for GADA, and 62%–76% and 98%–100% for IA-2A, respectively. In 2013–2020 the sensitivity and specificity were 32%–86% and 66%–88% for ICA. In 2020 sensitivity of 74% and a specificity of 100% was reported for the ZnT8A assay.

The strategy for islet autoantibody screening in the DIPP study is described in Table S1. In children born in 1994–2002 ICA were measured from every follow-up sample and if positive, IAA, GADA, and IA-2A were analyzed from all past and future samples of that child. In children born from January 2003 ICA, IAA, GADA, and IA-2A were analyzed regularly from every follow-up sample [6]. According to the revised DIPP Novum protocol launched in April 2019 IAA, GADA, IA-2A, and ZnT8A have been analyzed from every sample of every participant, and ICA was no longer analyzed. Retrospective analysis of IAA, GADA, IA-2A, and ZnT8A have been performed from the samples of the first 1,006 participants of the DIPP study born in 1994–1997 [7].

The ZnT8A method was established when DIPP study had already operated for more than a decade. In the DIPP study the aim has been to measure ZnT8A from serum samples of children with ≥2 other biochemical islet autoantibodies (IAA, GADA, and IA-2A). Thus, ZnT8A have mostly been measured retrospectively from available samples of these children. Altogether, ZnT8A have been analyzed in at least one sample in 80.9% of the children who had developed ≥2 other biochemical islet autoantibodies by March 2019. Since April 2019 ZnT8A have been measured from all DIPP follow-up samples. In addition, for the current study we analyzed ZnT8A from all available samples of children who had developed any other islet autoantibodies by the age of 0.50 years (Table S1).

### 2.3. Islet Autoantibodies in the TEDDY Study

IAA, GADA, and IA-2A for the Finnish TEDDY participants were analyzed from all follow-up samples since study inception with radiobinding assays in the University of Bristol, UK, and all positive samples were analyzed also in the Barbara Davis Center for Childhood Diabetes at the University of Colorado USA. These laboratories have also regularly participated in the DASP and IASP workshops with good performance for the assays. If the samples tested positive in both laboratories, the result was considered positive. ZnT8A were analyzed in all samples that were positive for IAA, GADA, or IA-2A [2].

### 2.4. Islet Autoantibody Positivity in the Current Analysis

For both DIPP and TEDDY children, we defined confirmed islet autoantibody positivity when the same islet autoantibody was positive in two consecutive samples, or if the child progressed to T1D shortly after the initial positivity. If the child became positive for any islet autoantibody in one sample, but the next sample was negative for that islet autoantibody, the positivity was considered transient.

### 2.5. HLA Genotyping

DNA was extracted from cord blood, the HLA gene segments to be analyzed were amplified with polymerase chain reaction, after which lanthanide labeled allele specific oligonucleotide probes were used for sequence specific hybridization. Detailed methods have been described earlier [6, 21, 22]. The DIPP screening procedure to define eligibility for follow-up initially included only a few risk-associated (DQB1 ^*∗*^ 02 and DQB1 ^*∗*^ 03 : 02) and protective (DQB1 ^*∗*^ 03 : 01 and DQB1 ^*∗*^ 06 : 02/03) alleles and was later expanded to cover more DQB1 alleles as well as DQA1 and DRB1 alleles informative for T1D risk definition [6]. The TEDDY study had more stringent inclusion criteria including only the highest risk HLA-genotypes as described earlier [2]. In this report we refer to the two major risk haplotypes as DR3-DQ2 (DQA1 ^*∗*^ 05-DQB1 ^*∗*^ 02) and DR4-DQ8 (DRB1 ^*∗*^ 04 : 01/2/4/5-DQA1 ^*∗*^ 03-DQB1 ^*∗*^ 03 : 02).

### 2.6. First-Degree Relatives, Season of Birth, Maternal Age at Birth

The information on T1D among first-degree relatives (FDR) (mother, father or sibling) was recorded at enrollment and updated at the follow-up visits in both the DIPP and TEDDY studies. Season of birth and maternal age at birth were only available for the participants in the DIPP study.

### 2.7. T1D Diagnosis

T1D was diagnosed according to World Health Organization and American Diabetes Association criteria [23, 24]. The diagnosis data of the DIPP study were available until February 2022 and diagnosis data of the TEDDY study were received from NIDDK Repository in May 2022.

### 2.8. Exclusion of Maternal Antibodies

**Figure 1:**
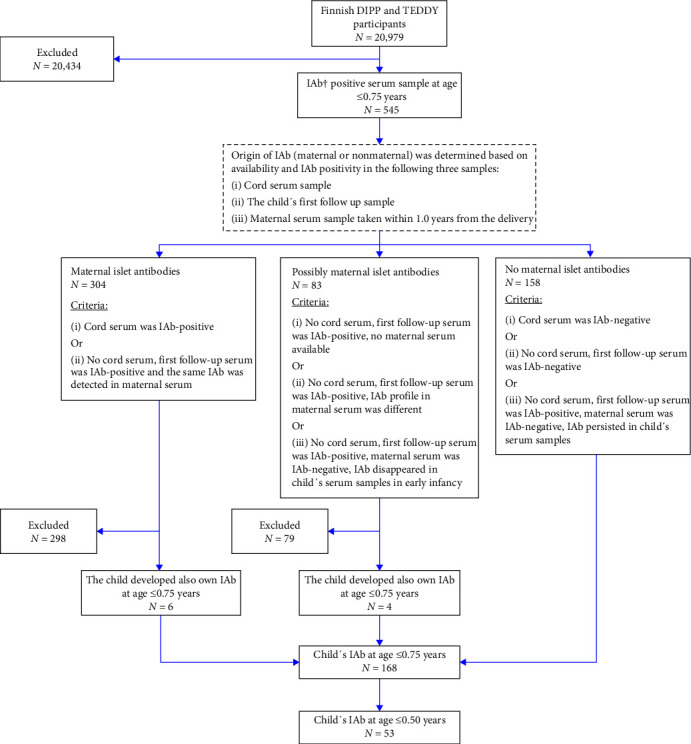
Exclusion of maternal islet antibodies. Description of the exclusion of maternal islet antibodies in 545 DIPP or TEDDY participants who had an islet autoantibody-positive sample at or before the age of 0.75 years. Any islet antibodies observed in the child at the age of ≤0.75 years were determined to be of either maternal origin, possibly maternal origin, or nonmaternal origin based on measurement of islet autoantibodies in cord serum sample, the child's first follow-up serum sample and the maternal serum sample taken during the 1st year after delivery. ^†^IAb = islet autoantibody.

We excluded maternally transferred islet antibodies by evaluating the child's first serum sample (cord or follow-up sample) and the child's later samples together with the maternal serum sample taken within 1 year after delivery (Figure 1). Autoantibodies were determined to be maternal if (1) the cord serum was positive for autoantibodies, (2) cord serum was not available but the child's first follow-up sample was positive for the same autoantibodies as the maternal serum sample. Autoantibodies were considered possibly maternal, but their origin could not be determined reliably if (1) the child's first follow-up sample was positive but neither cord nor maternal serum sample was available, (2) both the maternal sample and the first follow-up sample of the child were positive but the autoantibody profiles in these samples were different, (3) the first follow-up sample of the child was positive but maternal sample was negative and the positivity in the subsequent samples of the child disappeared. The child was determined not to have maternal autoantibodies if (1) their cord serum sample was negative, (2) in the absence of cord sample their first follow-up sample was negative, (3) in the absence of cord sample the first follow-up sample was positive, the maternal sample was negative, and the positivity in the child's subsequent samples persisted/spread (Figure 1).

### 2.9. Study Subjects

The study population of 20,979 consisted of children participating the DIPP follow-up by October 2019 and the Finnish participants of the TEDDY study. Among them, 545 tested positive for any islet autoantibody in serum sample taken at the age of 0.75 years or younger. Among them, 304 were determined to have maternally transferred antibodies. The origin of autoantibodies was determined to be possibly maternal in 83 children. The remaining 158 children did not have maternal antibodies and thus, had developed islet autoimmunity by themselves. In addition, 10 children with initially maternal or possibly maternal islet antibodies clearly developed also own islet autoantibodies by the age of 0.75 years. Consequently, a total of 168 children developed islet autoimmunity by the age of 0.75 years and among them, 53 by the age of 0.50 years (Figure 1).

We created four birth cohorts of the Finnish children participating in the DIPP or TEDDY follow-up. The first cohort included the first 1,006 DIPP participants with regular follow-up, born between November 1994 and July 1997, and DIPP participants who were born in January 2003–August 2004. Those who were born before 2003 and were not among the first 1,006 participants were excluded from the birth cohort comparison because islet autoantibody screening included only ICA measurement. The second cohort included children born during the recruitment period of the TEDDY study, from September 2004 to February 2010, and participated in either the DIPP or TEDDY follow-up. The third and fourth birth cohorts included children born between March 2010 and December 2014, and those born between January 2015 and July 2019, respectively. The third and fourth birth cohorts were determined to cover equally long time periods.

### 2.10. Statistical Analysis

Statistical analyses were executed with the IBM SPSS Statistics for Windows version 26. We used *χ^2^* test when comparing the groups for categorical variables, but if more than 20% of expected values were less than 5, Fisher's exact test was applied instead. The secular trend was analyzed with linear-by-linear test (IBM SPSS Statistics for Windows, version 29. Armonk, NY: IBM Corp). Mann Whitney *U*-test was used in group comparisons for non-normally distributed variables and independent samples *T* test for normally distributed variables. *P*-value less than 0.05 was considered significant.

## 3. Results

Among the 20,979 Finnish children with HLA-conferred increased risk for T1D participating in the DIPP or TEDDY study, 53 (25 girls, 47.2%) turned positive for any islet autoantibody by the age of 0.50 years. Altogether 168 (62 girls, 36.9%) developed at least one islet autoantibody by the age of 0.75 years.

### 3.1. Extremely Early Islet Autoimmunity by the Age of 0.50 Years

The characteristics of the 53 children positive for any islet autoantibody at the age of ≤0.50 years are presented in Table 1. During the follow-up, 31 (58.5%) developed confirmed positivity including eight (15.1%) children who were eventually diagnosed with T1D. Of the 53 children 22 (41.5%) were positive for one or more islet autoantibodies only transiently during the follow-up. The age at the first positive sample varied between 0.2 and 0.5 years. Altogether 17 (32.1%) of the 53 children had completed the follow-up (i.e., last visit at age ≥14.5 years or had progressed to T1D). The mean age at the latest follow-up visit of the 36 (67.9%) children who had not completed the follow-up was 7.0 years (1.1–13.1 years, SD 3.0 years), and of them 15 (28.3% out of the 53) were still in follow-up and 21 (39.6% out of the 53) were lost to follow-up (Table 1).

**Figure 2:**
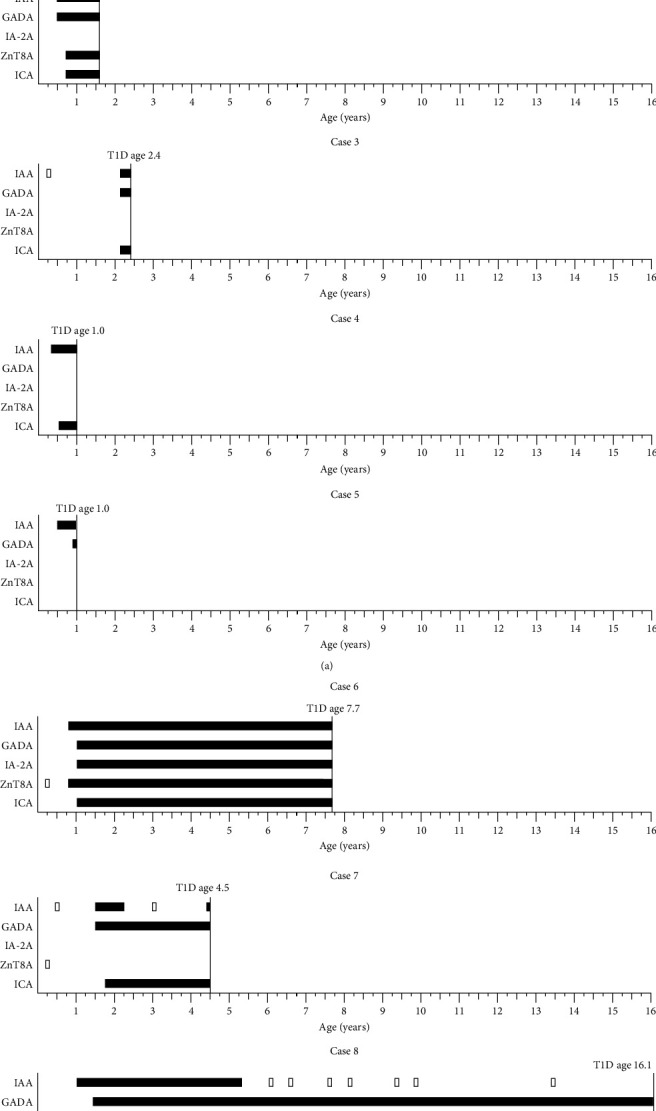
Islet autoantibody profiles of the progressors who developed autoantibodies by the age of 0.50 years. Solid black bar depicts confirmed positivity, empty bar with black outlines depicts a positive result, which was not confirmed in the following sample. Vertical line depicts the age at diagnosis of type 1 diabetes.

Islet autoantibody profiles of the children who developed islet autoimmunity by the age of 0.50 years and were eventually diagnosed with T1D are presented in Figure 2. All of them participated in the follow-up regularly until the diagnosis. None of them had any signs of rare autoimmune forms of diabetes. Among them the first islet autoantibody was either IAA alone or in combination, or ZnT8A alone. In the IAA-first group the age at diagnosis was very young, ≤2.4 years, while in the ZnT8A-first group the age at diagnosis varied remarkably between 4.5 and 16.1 years (Figure 2 and Table 1).

**Figure 3:**
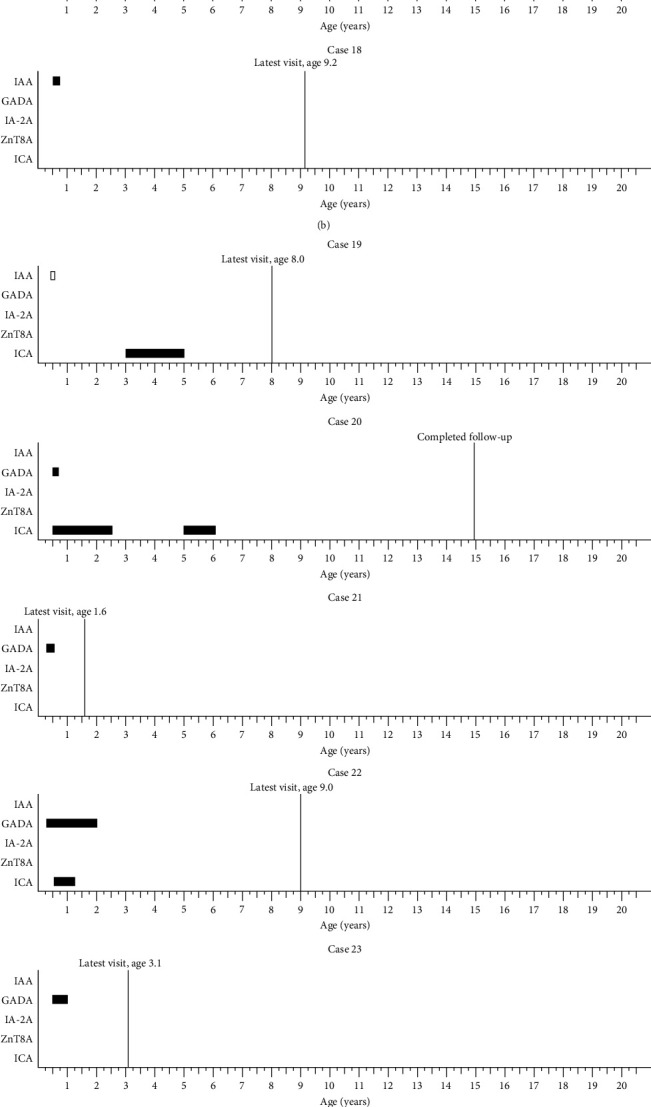
Islet autoantibody profiles of children who developed autoantibodies by the age of 0.50 years and progressed to confirmed positivity during follow-up. Solid black bar depicts confirmed positivity, empty bar with black outlines depicts a positive result, which was not confirmed in the following sample. Vertical line depicts the age at the latest follow-up visit.

Islet autoantibody profiles of the 23 children who developed islet autoimmunity by the age of 0.50 years and had confirmed positivity during the follow-up without progression to T1D are presented in Figure 3. Among them, IAA alone was the most common first appearing islet autoantibody observed in 11 (47.8%) children, while GADA was the first islet autoantibody either alone or with ICA in four (17.4%) children. The remaining eight (34.8%) children had ICA as the first single islet autoantibody. During the follow-up 16/23 (69.6%) had only one confirmed islet autoantibody whereas seven (30.4%) developed multipositivity. By the end of the follow-up 18/23 (78.3%) had become autoantibody negative (Figure 3 and Table 1). Among all 31 children with confirmed islet autoantibodies, including those who progressed to T1D, four (12.9%) had an FDR affected by T1D and seven (22.6%) carried the high-risk DR3-DQ2/DR4-DQ8 genotype (Table 1).

In the 22 children with only transient islet autoantibodies during follow-up, IAA and GADA were equally common as the first appearing islet autoantibodies. Two of them (9.1%) had an FDR with T1D and four (18.2%) carried the high-risk DR3-DQ2/DR4-DQ8 genotype (Table 1).

In the whole group of children with islet autoantibodies by the age of 0.50 years there were no significant differences in the frequencies of DR3-DQ2/DR4-DQ8 heterozygosity, DR4-DQ8-positive genotypes without the presence of DR3-DQ2, or DR3-DQ2-positive genotypes without DR4-DQ8 between the children who had single IAA, single GADA or single ZnT8A as their first appearing islet autoantibody (Table 2). The presence of DR4-DQ8 haplotype was equally common in children with IAA alone, GADA alone, or ZnT8A alone as the first appearing autoantibody (90.9%, 60.0%, and 100%, respectively, *p*=0.08) and the most common DR4 subtype was DRB1 ^*∗*^ 0401 (85.0%, 66.7%, and 100%, respectively, calculated from DR4-DQ8-positive subjects, *p*=0.47).

### 3.2. Islet Autoantibodies Appearing at the Age of 0.51–0.75 Years Compared to the Age of ≤0.50 Years

As expected, the appearance of islet autoantibodies became more frequent at the age of 0.51–0.75 years compared to the younger age (*N* = 115, 0.55% vs. *N* = 53, 0.25%). Children developing islet autoantibodies at the age of 0.51–0.75 years progressed more frequently to confirmed positivity than children who developed autoantibodies by the age of 0.50 years (81.7% vs. 58.5%, *p*=0.002). There were no significant differences between these groups in any other characteristics studied (Table 3). Also, when comparing only those who progressed to T1D, these characteristics did not differ (data not shown) and the age at diagnosis was similar (Table 3).

### 3.3. Season of Birth and Maternal Age at Birth in DIPP Children

Among the DIPP participants who developed islet autoantibodies by the age of 0.50 years, 19.2% were born in the winter months (December–February), 25.0% in spring (March–May), 36.6% in summer (June–August), and 19.2% in fall (September–November). There were no significant differences in season of birth, when children with islet autoantibodies by the age of 0.50 years and by the age of 0.75 years were compared to all other DIPP participants (data not shown). Among children who had islet autoantibodies by the age of 0.50 years, the mean maternal age at delivery was significantly older than in the rest of the DIPP participants (31.8 vs. 30.3 years; *p*=0.034). When considering all DIPP participants with islet autoantibodies by the age of 0.75 years, the mean maternal age was 30.9 years and the difference to rest of the DIPP population was no longer significant (*p*=0.16). The mean maternal age in the children developing islet autoantibodies by the age of 0.50 years and 0.51–0.75 years did not differ significantly (Table 3).

### 3.4. Secular Trend in Extremely Early Islet Autoimmunity

Children were compared in four birth cohorts including birth years 1994–1997 and 2003–2019. The proportion of children with any islet autoantibody by the age of 0.50 years decreased toward the most recent birth cohorts (0.6%, 0.2%, 0.5%, 0.1% in consecutive order, *p*=0.016). This difference remained significant when including only participants who had confirmed autoantibody positivity with or without progression to T1D during the follow-up (0.4%, 0.1%, 0.2%, 0.1% in consecutive order, *p*=0.018) (Table 4).

When extending the study population to children who developed islet autoantibodies by the age of 0.75 years, the result was similar in the group of children with any positivity (1.5%, 0.8%, 1.2%, 0.5% in consecutive order, *p*=0.009), and also when including only those participants who progressed to confirmed positivity during the follow-up (1.0%, 0.6%, 0.7%, 0.5%, *p*=0.048) (Table 4).

## 4. Discussion

In the combined data from the DIPP and TEDDY studies, we identified 53 Finnish children who developed extremely early islet autoimmunity before the age of 6 months. Among them, eight (15.1%) were eventually diagnosed with T1D and most often their first-appearing autoantibodies were IAA or ZnT8A. Progressors with IAA-initiated autoimmunity were diagnosed with clinical disease at a very young age between 1.0 and 2.4 years, while those with ZnT8A as the first-appearing autoantibody were diagnosed at an older and more variable age. We also identified a total of 115 children among whom islet autoantibodies appeared at the age of 6–9 months and during follow-up they developed more often confirmed positivity for islet autoantibodies than the 53 children with extremely early islet autoimmunity. Since our study cohort covered birth years from 1994 to 2019 we were also able to follow the secular trend of extremely early appearance of islet autoimmunity, which decreased significantly towards the most recent birth cohorts.

More than 20,000 Finnish children have participated in the DIPP or TEDDY prospective studies starting regular follow-up from the age of 3 months. The DIPP and TEDDY studies started newborn recruitment in Finland in 1994 and 2004, respectively, covering a period of more than 25 years. The current analysis was focused on birth years 1994–2019 and includes a large study population with the possibility to detect rare phenomena such as extremely early appearance of islet autoimmunity. Another strength of the study was the availability of the cord serum sample in the DIPP study and the maternal serum sample drawn soon after the birth in both the DIPP and TEDDY studies. Measurement of islet autoantibodies in the cord and/or maternal sample was essential to exclude the possibility that antibodies detected in the child's early follow-up sample were transferred from the mother. As a result, in our final study population of 53 children the maternal origin of islet antibodies was strictly excluded based on comparison of islet autoantibody profiles between the child's follow-up samples and the cord sample or maternal sample.

Our results showed that during the first months of life islet autoimmunity was most often initiated with IAA and that IAA-first was associated with rapid progression to clinical diabetes. These observations are in line with previous reports that IAA appears as the first islet autoantibody at a young age, and early appearance of IAA is associated with rapid progression to T1D [2, 5, 6]. Thus, children with extremely early IAA-initiated autoimmunity and subsequent progression to T1D depict one of the typical endotypes of T1D. Risk factors of the IAA-initiated and GADA-initiated T1D endotypes have been explored [25]. Interestingly, Coxsackievirus B infection has been reported to precede IAA-initiated autoimmunity [26] and could be a trigger for IAA-initiated disease process. None of the progressors in our study had single GADA as the first-appearing autoantibody which was not unexpected since the GADA-first endotype is known to appear at a later age than the IAA-first endotype [5, 6]. Less is known about ZnT8A as the first-appearing islet autoantibody. We identified children who initiated their islet autoimmunity with ZnT8A at very young age. These participants seemed to progress to T1D at a slower rate in comparison to those who started their disease process with IAA. These results expand the current view of islet autoimmunity emerging in early life, even though they should be interpreted with caution, as the numbers are very small. However, they are supported by an earlier study reporting that in newly diagnosed patients with T1D, positivity for ZnT8A increases by age [19]. In addition, an analysis combining data from other prospective study cohorts indicates that ZnT8A in the first multipositive sample is common among those who progress to T1D slowly [27].

The largest group of children with extremely early appearance of islet autoimmunity developed confirmed positivity during follow-up but did not progress to T1D. Three characteristics were typical for these participants. First, most of them (16/23) were positive for only one confirmed islet autoantibody. Second, in 6/7 children who had multiple autoantibodies the presence of ICA defined multipositivity together with one biochemical autoantibody. Third, most of these children (18/23) reverted to autoantibody negativity by the end of the follow-up. These findings are in line with earlier observations of children who are positive for single autoantibodies showing that the risk of progression to T1D is remarkably lower compared to multipositive individuals and lowest among those who revert to negativity during the first 2 years after initial seroconversion [1, 15, 28]. Furthermore, ICA in general is considered of less importance in prediction of the disease compared to other islet autoantibodies [7]. Interestingly, ZnT8A positivity was rare in this group of children, but this should be interpreted with caution, because the data on ZnT8A were not as complete as that on the other islet autoantibodies.

Transient islet autoantibody positivity was encountered relatively often, in 22/53 children with extremely early appearance of islet autoimmunity. Transient islet autoantibodies have frequently been observed also in older children participating in prospective follow-up [29]. In the present cohort the proportion of transient positivity was significantly more common in the group of children developing islet autoantibodies before the age of 6 months compared to those with first appearance of islet autoimmunity slightly later, at the age of 6–9 months (42% vs. 18%). Due to the ongoing follow-up, it is possible that some children who were observed to have only transient positivity at a very young age will later develop confirmed positivity for single or multiple autoantibodies. Fluctuation between positivity and negativity for islet autoantibodies may be observed before confirmed, persistent seroconversion, and in our study population of 53 children a couple of them were first transiently positive but later developed confirmed islet autoimmunity and even T1D. However, the 22 children categorized in the group of transient positivity had a relatively long follow-up until a mean age of 8.8 years suggesting that transient positivity was not indicating further progression of islet autoimmunity. Earlier studies have also indicated that transient islet autoimmunity is not associated with increased risk of future T1D [29, 30].

The majority of our study subjects with IAA-first were DRB1 ^*∗*^ 0401-positive, which is concurrent with the earlier studies reporting that IAA as the first-appearing islet autoantibody is associated with the HLA DR4-DQ8 haplotype whereas DR3-DQ2 is associated with GADA as the first autoantibody [6, 31, 32]. The DIPP study has recently analyzed the DR4-DQ8 positive cohort and reported that IAA was more often the first autoantibody in children with the DR4 subtype DRB1 ^*∗*^ 0401 than in those with DRB1 ^*∗*^ 0404 who had more often GADA as the first appearing autoantibody [33]. However, in the present small cohort of very young children such a difference could not be observed.

Pre- and perinatal factors are considered to play a crucial role in development of islet autoimmunity and T1D. The link between the season or month of birth and T1D has been studied with variable results. In the majority of the European areas, including Finland, no such association has been verified [34, 35], but in the northern states of the United States and in the United Kingdom birth in spring has been linked to the risk of T1D [34, 36, 37]. We did not find any significant differences in the season of birth between the DIPP children with extremely early autoimmunity compared to all other DIPP participants. Older maternal age has been reported to be associated with the risk of T1D in earlier studies [38], which is concurrent with our observation that the mothers of DIPP children with extremely early autoimmunity were older than those of other DIPP participants. These findings reinforce the role of prenatal environment as a contributing factor in the disease process. In our study the availability of information on maternal health and exposures was limited and future research should focus on these factors.

The incidence of T1D in Finland increased from the 1980s until 2006 whereafter it has plateaued. The rise in incidence was especially strong in the youngest age group of children <5 years of age [10, 11]. According to the latest report from Finland covering the majority of children with newly diagnosed T1D, the incidence of T1D had slightly decreased, mostly due to a decrease of the incidence in the youngest age group of children [39]. In line with this, we found that the incidence of extremely early islet autoimmunity decreased from the 1990s to 2010s. However, due to the small number of children in our study, these results should be interpreted with caution. Although none of the 53 children were diagnosed with T1D under the age of 12 months there is evidence that T1D can present even before the age of 6 months [40].

There are some limitations in our study. First, the assay for analyzing ZnT8A became available later than the methods for the other islet autoantibodies and therefore ZnT8A have been analyzed retrospectively from the children who became positive for ≥2 other islet autoantibodies (DIPP) or ≥1 autoantibody (TEDDY). In the DIPP study ZnT8A has also been analyzed from the first 1,006 participants regardless of positivity for other autoantibodies, giving systematic information on this group [7]. As we wanted to make the coverage of our analysis as complete as possible, we measured ZnT8A from all available samples of DIPP participants who were positive for any islet autoantibody by the age of 0.50 years. ZnT8A measurements were still not as complete as those for the other islet autoantibodies, and there may be a few children who participated in the DIPP or TEDDY follow-up and were positive for ZnT8A by the age of 0.50 years but were not identified. However, our data are an important addition to the current knowledge about the emergence of ZnT8A in young children and progression of the disease process initiated with ZnT8A. Future studies with more complete data on ZnT8A are needed to confirm these results. Second, 21/53 were lost to follow-up, with mean age of 6.25 years at the latest visit. Even though we do not have information on islet autoantibodies after the latest visit among these children, it is unlikely that we would have missed a participant diagnosed with T1D because the clinical diagnosis and treatment of T1D occur in the same three university hospitals where the DIPP and TEDDY studies operate. Furthermore, by the latest follow-up visit most of these children had reverted negative for islet autoantibodies, reflecting that their risk of T1D was not increased [1, 15]. Third, the origin of islet autoantibodies in a total of 83 children could not be determined indisputably due to the lack of both the cord and maternal serum sample or dissimilarity between the autoantibody profiles of the child's first positive sample and the maternal sample. However, islet antibodies determined as possibly maternal behaved usually like maternal islet antibodies, i.e., they had decreasing levels and disappeared in infancy. A few children in this group might have developed early islet autoantibodies themselves, which would have made our study group slightly larger. For clarity, however, we only analyzed children for whom we had evidence for their own islet autoantibody production and strictly excluded all participants possibly having maternally transferred antibodies. Fourth, we excluded the participants born in Aug 1997–Dec 2002 from the birth cohort analyses because during this time period, only ICA was used to screen islet autoimmunity, and only if it was positive, also IAA, GADA, and IA-2A were measured. Thus, among those children, there may be participants with extremely early islet autoimmunity whom we have not identified, and the comparison would have been less reliable. Finally, the assays of DIPP and TEDDY laboratories are not harmonized, which may affect the comparability of their results. However, the two TEDDY laboratories have harmonized their assays [41], and all DIPP and TEDDY laboratories have participated regularly in DASP and IASP workshops in order to monitor the performance of their assays.

## 5. Conclusions

We have demonstrated that the appearance of islet autoantibodies is rare but possible at a very early age of 6 months or younger. The most common first-appearing islet autoantibody at this age was IAA. Our data also show that children with extremely early seroconversion may rapidly progress to T1D. Interestingly, the pace of progression was rapid in children with IAA as the first appearing autoantibody but more variable in those with ZnT8A-first. We also demonstrated a temporal decline in the rate of extremely early seroconversions in Finnish children with increased HLA-conferred T1D risk. This study guides future research focusing on the first months of life with the aim to identify the triggers of islet autoimmunity and enable primary prevention of T1D.

## Figures and Tables

**Table 1:** Characteristics of 53 children with islet autoantibody positivity at or before the age of 0.50 years.

Case	First IAb^†^ (transient or confirmed)	Confirmed IAb	Age at latest visit or at diagnosis^‡^	Follow-up status	FDR present^§^	HLA Haplotype 1	HLA Haplotype 2
Cases progressing to T1D^¶^							
1	IAA, GADA, ICA	IAA, GADA, ICA	1.1	Completed	No	(DR3)–DQA1 ^*∗*^05–DQB1 ^*∗*^02	DRB1 ^*∗*^0404–DQA1 ^*∗*^03–DQB1 ^*∗*^0302
2	IAA, GADA	IAA, GADA, ZnT8A, ICA	1.6	Completed	No	DRB1 ^*∗*^0401–DQA1 ^*∗*^03–DQB1 ^*∗*^0302	(DR13)–DQB1 ^*∗*^0604
3	IAA	IAA, GADA, ICA	2.4	Completed	No	(DR3)–DQA1 ^*∗*^05–DQB1 ^*∗*^02	(DR3)–DQA1 ^*∗*^05–DQB1 ^*∗*^02
4	IAA	IAA, ICA	1.0	Completed	Sibling	(DR3)–DQA1 ^*∗*^05–DQB1 ^*∗*^02	DRB1 ^*∗*^0404–DQA1 ^*∗*^03–DQB1 ^*∗*^0302
5	IAA	IAA, GADA	1.0	Completed	No	DRB1 ^*∗*^0401–DQA1 ^*∗*^03–DQB1 ^*∗*^0302	(DR8)–DQB1 ^*∗*^04
6	ZnT8A	IAA, GADA, IA-2A, ZnT8A, ICA	7.7	Completed	No	(DR3)–DQA1 ^*∗*^05–DQB1 ^*∗*^02	DRB1 ^*∗*^0401–DQA1 ^*∗*^03–DQB1 ^*∗*^0302
7	ZnT8A	IAA, GADA, ICA	4.5	Completed	No	(DR3)–DQA1 ^*∗*^05–DQB1 ^*∗*^02	DRB1 ^*∗*^0401–DQA1 ^*∗*^03–DQB1 ^*∗*^0302
8	ZnT8A	IAA, GADA, IA-2A, ZnT8A, ICA	16.1	Completed	Sibling	DRB1 ^*∗*^0401–DQA1 ^*∗*^03–DQB1 ^*∗*^0302	(DR8)–DQB1 ^*∗*^04
Cases with confirmed positivity during follow-up							
9	IAA	IAA, ICA	15.0	Completed	No	DRB1 ^*∗*^0401–DQA1 ^*∗*^03–DQB1 ^*∗*^0302	(DR1/10)–DQB1 ^*∗*^0501
10	IAA	GADA, ICA	20.2	Completed	Father	DRB1 ^*∗*^0401–DQA1 ^*∗*^03–DQB1 ^*∗*^0302	(DR13)–DQB1 ^*∗*^0603
11	IAA	GADA	10.2	Lost to follow-up	No	DRB1 ^*∗*^0401–DQA1 ^*∗*^03–DQB1 ^*∗*^0302	DRB1 ^*∗*^0401–DQA1 ^*∗*^03–DQB1 ^*∗*^0302
12	IAA	IAA	6.9	Lost to follow-up	No	(DR3)–DQA1 ^*∗*^05–DQB1 ^*∗*^02	(DR9)–DQA1 ^*∗*^03–DQB1 ^*∗*^0303
13	IAA	IAA, GADA, IA-2A, ZnT8A	9.3	In follow-up	No	DRB1 ^*∗*^0404–DQA1 ^*∗*^03–DQB1 ^*∗*^0302	(DR3)–DQA1 ^*∗*^05–DQB1 ^*∗*^02
14	IAA	IAA	3.6	In follow-up	No	DRB1 ^*∗*^0401–DQA1 ^*∗*^03–DQB1 ^*∗*^0302	DRB1 ^*∗*^0401–DQA1 ^*∗*^03–DQB1 ^*∗*^0302
15	IAA	GADA	9.0	In follow-up	No	DRB1 ^*∗*^0401–DQA1 ^*∗*^03–DQB1 ^*∗*^0302	DRB1 ^*∗*^0404–DQA1 ^*∗*^03–DQB1 ^*∗*^0302
16	IAA	ICA	6.5	In follow-up	No	DRB1 ^*∗*^0401–DQA1 ^*∗*^03–DQB1 ^*∗*^0302	(DR9)–DQA1 ^*∗*^03–DQB1 ^*∗*^0303
17	IAA	IAA	6.0	In follow-up	No	(DR3)–DQA1 ^*∗*^05–DQB1 ^*∗*^02	DRB1 ^*∗*^0401–DQA1 ^*∗*^03–DQB1 ^*∗*^0302
18	IAA	IAA	9.2	In follow-up	No	DRB1 ^*∗*^0401–DQA1 ^*∗*^03–DQB1 ^*∗*^0302	(DR1/10)–DQB1 ^*∗*^0501
19	IAA	ICA	8.0	Lost to follow-up	No	DRB1 ^*∗*^0401–DQA1 ^*∗*^03–DQB1 ^*∗*^0302	DRB1 ^*∗*^0401–DQA1 ^*∗*^03–DQB1 ^*∗*^0302
20	GADA, ICA	GADA, ICA	14.9	Completed	No	DRB1 ^*∗*^0401–DQA1 ^*∗*^03–DQB1 ^*∗*^0302	(DR13)–DQB1 ^*∗*^0603
21	GADA	GADA	1.6	Lost to follow-up	No	(DR13)–DQB1 ^*∗*^0604	DRB1 ^*∗*^0403–DQA1 ^*∗*^03–DQB1 ^*∗*^0302
22	GADA	GADA, ICA	9.0	In follow-up	No	DRB1 ^*∗*^0401–DQA1 ^*∗*^03–DQB1 ^*∗*^0302	(DR8)–DQB1 ^*∗*^04
23	GADA	GADA	3.1	Lost to follow-up	No	(DR3)–DQA1 ^*∗*^05–DQB1 ^*∗*^02	(DR9)–DQA1 ^*∗*^03–DQB1 ^*∗*^0303
24	ICA	ICA	15.2	Completed	No	(DR3)–DQA1 ^*∗*^05–DQB1 ^*∗*^02	DRB1 ^*∗*^0404–DQA1 ^*∗*^03–DQB1 ^*∗*^0302
25	ICA	ICA	8.2	Lost to follow-up	No	DRB1 ^*∗*^0401–DQA1 ^*∗*^03–DQB1 ^*∗*^0302	(DR13)–DQB1 ^*∗*^0603
26	ICA	ICA	15.4	Completed	Mother	(DR15)–DQB1 ^*∗*^0602	DRB1 ^*∗*^0401–DQA1 ^*∗*^03–DQB1 ^*∗*^0302
27	ICA	IAA, ICA	1.1	Lost to follow-up	No	(DR7)–DQA1 ^*∗*^0201–DQB1 ^*∗*^02	DRB1 ^*∗*^0401–DQA1 ^*∗*^03–DQB1 ^*∗*^0302
28	ICA	IAA, ICA	3.1	Lost to follow-up	No	DRB1 ^*∗*^0401–DQA1 ^*∗*^03–DQB1 ^*∗*^0302	(DR1/10)–DQB1 ^*∗*^0501
29	ICA	ICA	4.0	Lost to follow-up	No	DRB1 ^*∗*^0404–DQA1 ^*∗*^03–DQB1 ^*∗*^0302	(DR1/10)–DQB1 ^*∗*^0501
30	ICA	ICA	15.0	Completed	No	DRB1 ^*∗*^0401–DQA1 ^*∗*^03–DQB1 ^*∗*^0302	(DR9)–DQA1 ^*∗*^03–DQB1 ^*∗*^0303
31	ICA	ICA	5.0	Lost to follow-up	No	DRB1 ^*∗*^0404–DQA1 ^*∗*^03–DQB1 ^*∗*^0302	(DR1/10)–DQB1 ^*∗*^0501
Cases with transient positivity only							
32	IAA		15.0	Completed	No	DRB1 ^*∗*^0401–DQA1 ^*∗*^03–DQB1 ^*∗*^0302	(DR9)–DQA1 ^*∗*^03–DQB1 ^*∗*^0303
33	IAA		7.1	Lost to follow-up	No	DRB1 ^*∗*^0401–DQA1 ^*∗*^03–DQB1 ^*∗*^0302	(DR13)–DQB1 ^*∗*^0603
34	IAA		15.7	Completed	No	(DR3)–DQA1 ^*∗*^05–DQB1 ^*∗*^02	DRB1 ^*∗*^0404–DQA1 ^*∗*^03–DQB1 ^*∗*^0302
35	IAA		6.8	Lost to follow-up	No	DRB1 ^*∗*^0401–DQA1 ^*∗*^03–DQB1 ^*∗*^0302	(DR1/10)–DQB1 ^*∗*^0501
36	IAA		2.0	Lost to follow-up	No	DRB1 ^*∗*^0401–DQA1 ^*∗*^03–DQB1 ^*∗*^0302	(DR8)–DQB1 ^*∗*^04
37	IAA		6.2	In follow-up	No	DRB1 ^*∗*^0401–DQA1 ^*∗*^03–DQB1 ^*∗*^0302	(DR8)–DQB1 ^*∗*^04
38	IAA		3.9	Lost to follow-up	No	(DR3)–DQA1 ^*∗*^05–DQB1 ^*∗*^02	DRB1 ^*∗*^0401–DQA1 ^*∗*^03–DQB1 ^*∗*^0302
39	IAA		8.9	In follow-up	No	DRB1 ^*∗*^0401–DQA1 ^*∗*^03–DQB1 ^*∗*^0302	(DR8)–DQB1 ^*∗*^04
40	GADA, ICA		7.3	In follow-up	No	DRB1 ^*∗*^0401–DQA1 ^*∗*^03–DQB1 ^*∗*^0302	(DR8)–DQB1 ^*∗*^04
41	GADA		9.0	In follow-up	Mother	DRB1 ^*∗*^0401–DQA1 ^*∗*^03–DQB1 ^*∗*^0302	(DR1/10)–DQB1 ^*∗*^0501
42	GADA		3.9	Lost to follow-up	No	DRB1 ^*∗*^0401–DQA1 ^*∗*^03–DQB1 ^*∗*^0302	(DR1/10)–DQB1 ^*∗*^0501
43	GADA		10.4	Lost to follow-up	No	(DR3)–DQA1 ^*∗*^05–DQB1 ^*∗*^02	(DR8)–DQB1 ^*∗*^04
44	GADA		6.5	Lost to follow-up	No	(DR3)–DQA1 ^*∗*^05–DQB1 ^*∗*^02	DRB1 ^*∗*^0404–DQA1 ^*∗*^03–DQB1 ^*∗*^0302
45	GADA		15.0	Completed	No	DRB1 ^*∗*^0404–DQA1 ^*∗*^03–DQB1 ^*∗*^0302	(DR1/10)–DQB1 ^*∗*^0501
46	GADA		13.1	Lost to follow-up	No	(DR3)–DQA1 ^*∗*^05–DQB1 ^*∗*^02	(DR14)–DQB1 ^*∗*^0503
47	GADA		5.9	Lost to follow-up	No	DRB1 ^*∗*^0401–DQA1 ^*∗*^03–DQB1 ^*∗*^0302	(DR13)–DQB1 ^*∗*^0604
48	ZnT8A		9.5	Lost to follow-up	No	(DR3)–DQA1 ^*∗*^05–DQB1 ^*∗*^02	DRB1 ^*∗*^0401–DQA1 ^*∗*^03–DQB1 ^*∗*^0302
49	IA-2A		11.1	Lost to follow-up	Sibling	(DR7)–DQA1 ^*∗*^0201–DQB1 ^*∗*^02	DRB1 ^*∗*^0401–DQA1 ^*∗*^03–DQB1 ^*∗*^0302
50	IA-2A		5.9	In follow-up	No	DRB1 ^*∗*^0401–DQA1 ^*∗*^03–DQB1 ^*∗*^0302	(DR8)–DQB1 ^*∗*^04
51	IA-2A		9.1	In follow-up	No	DRB1 ^*∗*^0401–DQA1 ^*∗*^03–DQB1 ^*∗*^0302	DRB1 ^*∗*^0404–DQA1 ^*∗*^03–DQB1 ^*∗*^0302
52	ICA		9.0	In follow-up	No	(DR7)–DQA1 ^*∗*^0201–DQB1 ^*∗*^02	(DR3)–DQA1 ^*∗*^05–DQB1 ^*∗*^02
53	ICA		12.1	In follow-up	No	DRB1 ^*∗*^0401–DQA1 ^*∗*^03–DQB1 ^*∗*^0302	(DR13)–DQB1 ^*∗*^0603

*Note*: The children are presented in three groups, first those who were diagnosed with type 1 diabetes during the follow-up, second those who had confirmed positivity for some islet autoantibody/-ies during the follow-up, and third those who were only transiently positive for any islet autoantibody during the follow-up. Each group is organized according to the first appearing islet autoantibody. The ages are given in years. ^†^Islet autoantibody. ^‡^Diagnosis of type 1 diabetes. ^§^First-degree relative with type 1 diabetes present. ^¶^Type 1 diabetes.

**Table 2:** Frequencies of HLA genotypes according to the first-appearing islet autoantibody in children with extremely early islet autoimmunity by the age of 0.50 years.

	IAA only first, *N* (%)	GADA only first, *N* (%)	ZnT8A only first, *N* (%)	*p*-Value
DR3-DQ2/DR4-DQ8	5 (22.7%)	1 (10.0%)	3 (75.0%)	0.053
DR4-DQ8/non-DR3-DQ2	15 (68.2%)	5 (50.0%)	1 (25.0%)	0.26
DR3-DQ2/non-DR4-DQ8	2 (9.1%)	3 (30.0%)	0 (0.0%)	0.27

*Note*: Frequencies of children who had HLA DR3-DQ2/DR4-DQ8 heterozygosity, a genotype with DR4-DQ8 but without DR3-DQ2, or a genotype with DR3-DQ2 but without DR4-DQ8 in three groups with either insulin autoantibodies (IAA), autoantibodies against glutamic acid decarboxylase (GADA), or antibodies against zinc transporter 8 (ZnT8A) appearing as the only first-appearing autoantibody, either confirmed or transient, by the age of 0.50 years. Fisher's exact test was used to compare the groups. (DRB1 ^*∗*^0403 was not included in the DR4-DQ8 haplotype).

**Table 3:** Comparison between children who developed islet autoantibodies either at the age of 0.51–0.75 years or by the age of 0.50 years.

	Islet autoantibodies at age 0.51–0.75 years	Islet autoantibodies at age ≤0.50 years	*p*-Value
	*N* = 115	*N* = 53	
Girls, *N* (%)	37 (32.2%)	25 (47.2%)	0.09
FDR^§^ with T1D^†^, *N* (%)	12 (10.4%)	6 (11.3%)	1.00
HLA DR3/DR4, *N* (%)	27 (26.2%)	11 (20.8%)	0.56
HLA DR4 without DR3, *N* (%)	63 (61.2%)	35 (66.0%)	0.60
HLA DR3 without DR4, *N* (%)	13 (12.6%)	6 (11.3%)	1.00
Maternal age at birth^‡^ (mean, in years)	30.4	31.8	0.13
Only transient islet autoantibodies, *N* (%)	21 (18.3%)	22 (41.5%)	0.002
Confirmed islet autoantibodies, *N* (%)	94 (81.7%)	31 (58.5%)	0.002
≥2 confirmed islet autoantibodies, *N* (%)	49 (42.6%)	15 (28.3%)	0.09
IAA only first, *N* (%)	47 (40.9%)	22 (41.5%)	1.00
GADA only first, *N* (%)	20 (17.4%)	10 (18.9%)	0.83
IA-2A only first, *N* (%)	3 (2.6%)	3 (5.7%)	0.38 ^*∗*^
Multiple islet autoantibodies first, *N* (%)	8 (7.0%)	4 (7.5%)	1.0
Completed follow-up, *N* (%)	42 (36.5%)	17 (32.1%)	0.61
T1D^†^, *N* (%)	26 (22.6%)	8 (15.1%)	0.31
Age at diagnosis (median, in years)	2.68	2.00	0.63

*Note*: Chi-square or Fisher´s exact ^*∗*^ tests were used to compare difference in frequencies. Mann Whitney *U*-test was used to compare the median ages and independent samples *T*-test to compare the mean age. Complete HLA was unavailable for 12 subjects. Follow-up was completed if participant had been diagnosed with T1D or participated the follow-up until the age of 14.50 years. ^§^First-degree relative. ^†^Type 1 diabetes. ^‡^Not available for TEDDY participants.

**Table 4:** Birth cohort comparison of children developing islet autoimmunity by the age of 0.50 years or by the age of 0.75 years.

	Birth cohorts				*p*-Value
	I	II	III	IV	
	*N* = 2998	*N* = 4782	*N* = 3159	*N* = 2734	
IAb^†^ at age ≤0.50 years, *N* (%)	19 (0.6%)	10 (0.2%)	15 (0.5%)	3 (0.1%)	0.016
IAb at age ≤0.50 years and confirmed positivity during follow-up, *N* (%)	12 (0.4%)	6 (0.1%)	6 (0.2%)	2 (0.1%)	0.018
IAb at age ≤0.75 years, *N* (%)	44 (1.5%)	39 (0.8%)	38 (1.2%)	15 (0.5%)	0.009
IAb at age ≤0.75 years and confirmed positivity during follow-up, *N* (%)	30 (1.0%)	29 (0.6%)	23 (0.7%)	13 (0.5%)	0.048

*Note*: The frequencies of children developing islet autoantibodies by the age of 0.50 years or by the age of 0.75 years and frequencies of those who developed confirmed islet autoantibody positivity during the follow-up. The four birth cohorts were defined according to the date of birth: I: November 1994–July 1997 and January 2003–August 2004, II: September 2004–February 2010, III: March 2010–December 2014, IV: January 2015–July 2019. Differences in the frequencies between the four cohorts were analyzed with linear-by-linear test (IBM SPSS Statistics for Windows, version 29. Armonk, NY: IBM Corp.). ^†^Islet autoantibody.

## Data Availability

The data set analyzed in this study is combined from the DIPP and TEDDY studies. The DIPP study data are not publicly available, but they can be available from the corresponding author with reasonable request. The TEDDY data ((V28)/https://doi.org/10.58020/y3jk-x087) are available on NIDDK Repository on request.

## Abbreviations

DASP: Diabetes Islet Autoantibody Standardization Program
DIPP: Type 1 Diabetes Prediction and Prevention Study
FDR: First-degree relative
GADA: Glutamic acid decarboxylase autoantibodies
HLA: Human leukocyte antigen
IAA: Insulin autoantibodies
IA-2A: Insulinoma-associated antigen-2 autoantibodies
ICA: Islet cell antibodies
IASP: Islet Autoantibody Standardization Program
NIDDK: National Institute of Diabetes and Digestive and Kidney Diseases
TEDDY: The Environmental Determinants of Diabetes in the Young study
T1D: Type 1 diabetes
ZnT8A: Zinc transporter 8 autoantibodies.

## Additional Points

*Preregistration of Studies*. Both the Type 1 Diabetes Prediction and Prevention Study (DIPP) and the Environmental Determinants of Diabetes in the Young study (TEDDY) have been registered in ClinicalTrials.gov, identifiers: NCT03269084 and NCT00279318, respectively.

## Supplementary Materials

Supplementary Materials Table S1: the autoantibody analyses performed in the DIPP study since 1994.
