# Supplementary material for: Extremely Early Appearance of Islet Autoantibodies in Genetically Susceptible Children
Source: Pediatr Diabetes. 2023 Dec 11;2023:9973135. doi: 10.1155/2023/9973135 (PMC12017086; doi:10.1155/2023/9973135)
Supplement: Supplementary Materials — Table S1: the autoantibody analyses performed in the DIPP study since 1994. [file 9973135.f1.docx]

| **Autoantibody screening in DIPP follow-up protocol** | |
| --- | --- |
| Children born Nov, 1994 – Dec, 2002 | ICA was analyzed from every sample. When ICA was positive, also IAA, GADA and IA-2A were analyzed from all past and future samples of the child. |
| Children born Jan, 2003 – Mar, 2019 | ICA, IAA, GADA and IA-2A and were analyzed from every sample. |
| All follow-up samples since Apr, 2019 | IAA, GADA, IA-2A and ZnT8A analyzed from every sample. Including participants already in the protocol and all new participants born Apr, 2019 onwards. |
| **Retrospective autoantibody testing** | |
| - IAA, GADA, IA-2A and ZnT8A were analyzed systematically from the samples of the 1006 first participants of the DIPP study, who were born Nov, 1994 – Jul, 1997. - ZnT8A were analyzed from the samples of children with positivity to ≥2 biochemical islet autoantibodies (IAA, GADA, IA-2A). - ZnT8A were analyzed from all children who developed any other islet autoantibodies by age 0.50 years. | |

**Supplementary Table 1.**
